# Immunomodulation of human intestinal T cells by the synthetic CD80 antagonist RhuDex®

**DOI:** 10.1002/iid3.34

**Published:** 2014-11-03

**Authors:** Anne-Kristin Heninger, Sabine Wentrup, Mohammed Al-Saeedi, Serin Schiessling, Thomas Giese, Florian Wartha, Stefan Meuer, Jutta Schröder-Braunstein

**Affiliations:** 1 Institute for Immunology, University Hospital HeidelbergIm Neuenheimer Feld 305, 69120, Heidelberg, Germany; 2 Department of General, Visceral and Transplant Surgery, University Hospital HeidelbergIm Neuenheimer Feld 110, 69120, Heidelberg, Germany; 3 Medigene AGLochhamer Str. 11, 82152, Planegg/Martinsried, Germany

**Keywords:** CD80 blockage, immunomodulation, inflammatory bowel disease, mucosal immunology, T cells

## Abstract

Deregulated activation of mucosal lamina propria T cells plays a central role in the pathogenesis of intestinal inflammation. One of the means to attenuate T cell activation is by blocking the CD28/CD80 co-stimulatory pathway. Here we investigate RhuDex®, a small molecule that binds to human CD80, for its effects on the activation of lamina propria T cells employing a gut-culture model of inflammation. To this end, lamina propria leukocytes (LPL) and peripheral blood lymphocytes (PBL) were stimulated either through the CD3/T-cell-receptor complex or the CD2-receptor (CD2) employing agonistic monoclonal antibodies. Co-stimulatory signals were provided by CD80/CD86 present on lamina propria myeloid cells or LPS-activated peripheral blood monocytes. Results show that RhuDex® caused a profound reduction of LPL and PBL proliferation, while Abatacept (CTLA-4-Ig) inhibited LPL proliferation to a small degree, and had no effect on PBL proliferation. Furthermore, Abatacept significantly inhibited IL-2, TNF-α, and IFN-γ release from LPL, primarily produced by CD4^+^ T cells, where IL-2 blockage was surprisingly strong, suggesting a down-regulating effect on regulatory T cells. In contrast, in the presence of RhuDex®, secretion of IL-17, again mostly by CD4^+^ T cells, and IFN-γ was inhibited in LPL and PBL, yet IL-2 remained unaffected. Thus, RhuDex® efficiently inhibited lamina propria and peripheral blood T-cell activation in this pre-clinical study making it a promising drug candidate for the treatment of intestinal inflammation.

## Introduction

Inflammatory bowel diseases (IBD), such as ulcerative colitis and Crohn's disease, represent major disorders of the human gastrointestinal tract. On the molecular level, IBD is characterized by an aberrant immune response against the commensal gut flora in genetically susceptible hosts. To date, drug treatment of IBD is mostly restricted to broadly active anti-inflammatory compounds such as glucocorticoids and 5-aminosalicylic acid (5-ASA). The more specific application of therapeutic monoclonal antibodies inhibiting TNF-α is successful only in a subset of patients, can be associated with severe side effects, and may only be effective for a short time frame [1,2]. Hence, in chronic IBD there exists a definite need for novel selective immunomodulatory drugs that are well tolerated by patients, can be administered orally, and long-term.

T cells constitute a prominent immune cell population in the intestinal mucosa. Functionally, T cells of the healthy intestinal immune system are characterized by a low proliferative response to anti-CD3/T cell receptor (TCR) stimulation when compared to peripheral blood T cells [3]. Yet, at the same time, lamina propria T cells sensitively respond to CD2 ligation, an antigen-independent pathway of T cell activation [4–6], or CD28 co-stimulation, producing significantly higher amounts of cytokines than their peripheral blood counterparts [7,8]. Importantly, a dysregulation of T cell responses has been observed in IBD. For example, effector T cells isolated from inflamed mucosal specimens of Crohn's disease patients show an enhanced reactivity to anti-CD3/TCR stimulation when compared to T cells obtained from normal (non-inflamed) gut mucosa [9,10]. Furthermore, expression of the CD28 ligands CD80 and CD86, which is not detectable in the intestinal mucosa under homeostatic conditions, is up-regulated on lamina propria myeloid cells in IBD [11]. Based on these observations, compounds that target and inhibit T cell activation and proliferation, for example by interfering with the CD28–CD80/CD86 co-stimulatory pathway, represent promising drug candidates for the treatment of IBD. Here, we explored the effects of RhuDex®, a small molecule that binds specifically to human CD80 and blocks T cell activation, proliferation and the secretion of cytokines [12]. The influence of RhuDex® on lamina propria T cell activation was investigated employing an ex-vivo human organ culture model. In this model, EDTA-mediated loss of the epithelial layer initiates an inflammatory response in resident lamina propria cells of normal mucosa, which shows many features of inflammation as are observed also in IBD patients [13]. Of note, the expression of CD80 (and CD86) is induced in lamina propria myeloid cells under these conditions. Importantly, this model allowed a standardized setting to test RhuDex® in the absence of immunosuppressive or anti-inflammatory medications as taken by IBD patients. The effect of RhuDex® on lamina propria T cells, as compared to peripheral blood T cells (autologous and allogeneic), stimulated through the TCR (via anti-CD3 antibody) or the CD2-receptor (via anti-CD2 antibodies) was studied with regard to cytokine production and proliferation. For comparison, another inhibitor of co-stimulation through CD28, the immunomodulatory drug Abatacept (CTLA-4-Ig) was employed [14]. In this model, RhuDex® was shown to be an inhibitor of T cell proliferation and the secretion of IL-17 and IFN-γ in lamina propria and peripheral blood T cells.

## Research Design and Methods

### Human subjects and samples

Colonic tissue from 7 individuals (numbered 1–7, Table S1) undergoing resection for localized colon cancer or benign colonic diseases plus autologous peripheral venous blood (PB) from four of those individuals were collected with informed consent and approval of the University of Heidelberg ethics committee (ethics vote number: 024/2003). As a control, PB from four healthy adults (numbered I–IV) was also collected. The study was performed in accordance with the principles laid down in the Declaration of Helsinki. The mucosa (approximately 50 cm^2^), removed from fresh surgical colonic tissue, was assessed by a pathologist to be free from any detectable pathologic changes as judged by microscopy. Subsequently, this mucosa tissue sample was immediately processed for setting up the organ culture model (LEL model, see below). The median age of healthy blood donors was 34 years (interquartile rage 30–36 years), and of tissue/autologous blood donors was 67 years (interquartile rage 63–75 years).

### PBL isolation

PB was collected in sodium-heparin, and peripheral blood mononuclear cells (PBMC) were isolated by density centrifugation over Ficoll–Hypaque. PBMC were split as follows: one fraction was incubated in culture medium (RPMI 1640 supplemented with 10% FCS, 2 mM Glutamine, 100 Units/mL Penicillin and Streptomycin) for 8 h to allow for plastic adherence. Subsequently, non-adherent peripheral blood lymphocytes (PBL) were collected for application in the T cell stimulation assay. Isolation of CD14^+^ monocytes from the other PBMC fraction was achieved by MACS negative isolation according to manufacturer's instructions (Monocyte Isolation Kit II; Miltenyi Biotech, Cologne, Germany). The purity of isolated monocytes (92.7 ± 3.8%) was confirmed by CD14 and CD33 staining. For the induction of CD80 expression, monocytes were activated with 1 µg/mL LPS (Sigma–Aldrich, St. Louis, MO, USA) for 8 h and subsequently washed three times in PBS before application in the T cell stimulation assay.

### LEL (loss of epithelial layer) model of intestinal inflammation

The organ culture was performed as previously described [15]. First, the whole mucosa of healthy human colonic tissue was washed extensively in RPMI 1640 + antibiotics (100 Units/mL Penicillin and Streptomycin, 2.5 µg/mL Amphotericin B, 10 µg/mL Ciprobay, 50 µg/mL Gentamicin, 96 µg/mL Cotrimoxazol). Next, mucus was removed by shaking the mucosa on an orbital shaker for 15 min in HBSS + antibiotics (as specified above) supplemented with 1 mM DTT (Sigma–Aldrich). Subsequently, the epithelial cell layer was removed by treating the mucosa with 0.7 mM EDTA (Sigma–Aldrich) for 30 min followed by washing in HBSS + antibiotics (as specified above). This exposure to EDTA is the important step to start the activation process of resident lamina propria cells. The EDTA/washing procedure was performed three times. Finally, the mucosa was placed in tissue culture dishes and incubated (37°C, 7.0% CO_2_) in culture medium + antibiotics (3.5 mL/cm^2^ of mucosa area). After 33–36 h, lamina propria leukocytes, that had migrated into the medium (Walk-Out (WO)-LPL), were harvested and the mucosa was discarded. Finally, WO-LPL were washed, resuspended in culture medium and allowed to rest for 30 min at 4°C before application in the T cell stimulation assay.

### Cell staining and flow cytometry

The following anti-human monoclonal antibodies were used for FACS cell surface staining. BD Biosciences (Heidelberg, Germany): CD25 APC-H7 (M-A251), CD3 FITC (SK7), CD3 V450 (SP34-2), CD33 PE-Cy7 (P67.6) or CD33 APC (WM53), CD80 PE (L307.4), CD86 Alexa Flour 700 (2331), CD14 FITC (MϕP9), CD66b Alexa Flour 647 (G10F5), HLA-DR V500 (G46-6) and the Annexin V FITC apoptosis detection kit I; eBioscience (San Diego, CA, USA): CD19 PerCP-Cy5.5 (HIB19); BioLegend (San Diego, CA, USA): CD14 Brilliant Violet 570 (M5E2). After staining, cells were washed twice with FACS buffer. Cells were acquired on a Becton Dickinson LSRII flow cytometer with FACS Diva software version 7.0. Doublets and clumps were excluded based on SSC-A versus SSC-W plots. Live cell populations were gated as 7-AAD (BD Biosciences) negative cells. CD66b was used to exclude eosinophils. At least 30,000 gated events were acquired for each sample and analyzed using FlowJo software version 9.3.2.

### Inhibitors

The synthetic CD80 antagonist RhuDex® (kindly provided from Medigene AG, Martinsried, Germany) was stored at 4°C. For each experiment, powderous RhuDex® choline salt was dissolved in H_2_O to obtain a stock concentration of 10 µg/mL RhuDex® free acid. All mentioned concentrations of RhuDex® always refer to the active moiety free acid, into which the choline salt dissociates in physiological media. Abatacept (Orencia®, Bristol-Myers Squibb GmbH & Co. KGaA, Munich, Germany) was reconstituted in PBS to the same stock concentration as RhuDex® and subsequently filter sterilized, aliquoted, and frozen at −80°C. For comparison, a blocking mouse monoclonal antibody (mAb) against human CD80 (IgG1; clone 2D10, BioLegend) was used in some assays [16].

### T cell stimulation assay

LPS-activated blood monocytes were plated at 10,000 cells/well and non-adhered PBL were immediately seeded on top at 100,000 cells/well in 96-well plates. WO-LPL were plated at 110,000 cells/well. Next, the inhibitors were quickly added to obtain a final concentration of 1 and 10 µg/mL Abatacept or 0.5, 3, and 20 µg/mL RhuDex® or 5 and 0.5 µg/mL anti-CD80 antibody, where indicated. T cells were stimulated with monoclonal antibodies (produced in house [17]) as follows: either by plate-bound anti-CD3 (OKT3, 0.03 µg/mL), or by a mixture of the three soluble anti-CD2 stimulating antibodies M1, M2 (both 0.5 µg/mL), and 3PT (0.33 µg/mL). Allogeneic blood was collected one day after colon resection surgery, treated the same way as autologous blood and set up in the stimulation assay simultaneously with WO-LPL. All cells were cultured at 37°C, 7% CO_2_.

To determine the effect of RhuDex on proliferation in a culture system lacking the presence of CD80, the Jurkat T cell line was used. To this end, 50,000 or 25,000 Jurkat T cells/well were pipetted into a 96-well plate, and RhuDex® or Abatacept were added at the beginning of culture. Cells were incubated for 20 h in the presence of 1 µCi/well ^3^[H]-thymidine.

### Cytokine measurements

Culture supernatants from the stimulation assay were collected after 24 h of incubation. The Cytometric Bead Array (CBA, BD Bioscience, Heidelberg, Germany) kit with Enhanced Sensitivity Flex Sets (IL-17, IL-2, IFN-γ, and TNF-α) was employed to quantify cytokine concentrations according to manufacturer's protocol. The assay detection range was between 0.274 and 200 pg/mL. Standard curves and samples were measured in technical duplicates on a LSRII flow cytometer and analyzed with FCAP ArrayTM v1.0.1 software (BD Bioscience). To detect T cell specific cytokine production, cells were stimulated as described above. After 2 h of incubation, 10 µg/mL Brefeldin A (Sigma–Aldrich) was added for an additional 4 h. Subsequently, cells were harvested, pooling two wells per condition, and the intracellular staining procedure was performed using BD Cytofix/Cytoperm™ (BD Biosciences) solutions according to manufacturer's instructions. After permeabilization, cells were stained for 30 min with IFN-γ FITC (clone 25723.11), IL-2 APC (clone 5344.11), TNF-α PE (cloneMAb11), CD3 V500, CD4 Pacific Blue, CD8 Alexa Flour 700 (all from BD Bioscience) or IL-17A PE (clone eBio64DEC17, eBioscience). Cells were analyzed using a Becton Dickinson LSRII flow cytometer acquiring 50,000 CD3^+^ T cells for each sample.

### Methyl-^3^[H]-thymidine incorporation assay

To assess proliferation, ^3^[H]-thymidine (1 µCi/well) was added for the last 16–18 h of incubation in the stimulation assay. Subsequently, cells were automatically harvested with a Tomtec 96 Harvester and collected onto a 96-well 1.2 µm pore-size filter-plate. ^3^[H]-thymidine incorporation was measured as counts per minute (cpm) using a Top Count Microplate Scintillation beta-particle counter.

### Statistical analysis

Results are presented as mean and standard deviation (SD). Expression of surface molecules on cell subsets was determined as percentage (%) of the indicated parent cell population. Expression of intracellular cytokines are reported as percentage (%) of CD3^+^CD4^+^ or CD3^+^CD8^+^ T cell parent populations. The mean responses of each donor in the stimulation assay were normalized by setting responses without inhibitors to 100%, and calculating responses in the presence of inhibitors accordingly. For normally distributed data, the one-way ANOVA and Dunnett's multiple comparisons test were applied to compare means of the same subject tested under different conditions. For not normally distributed data, the Friedman test was performed with Dunn's multiple comparisons test. For all tests, a two-tailed *P* value of <0.05 was considered to be significant.

## Results

### Presence of CD80 and CD86 in the assay system

**Figure 1:**
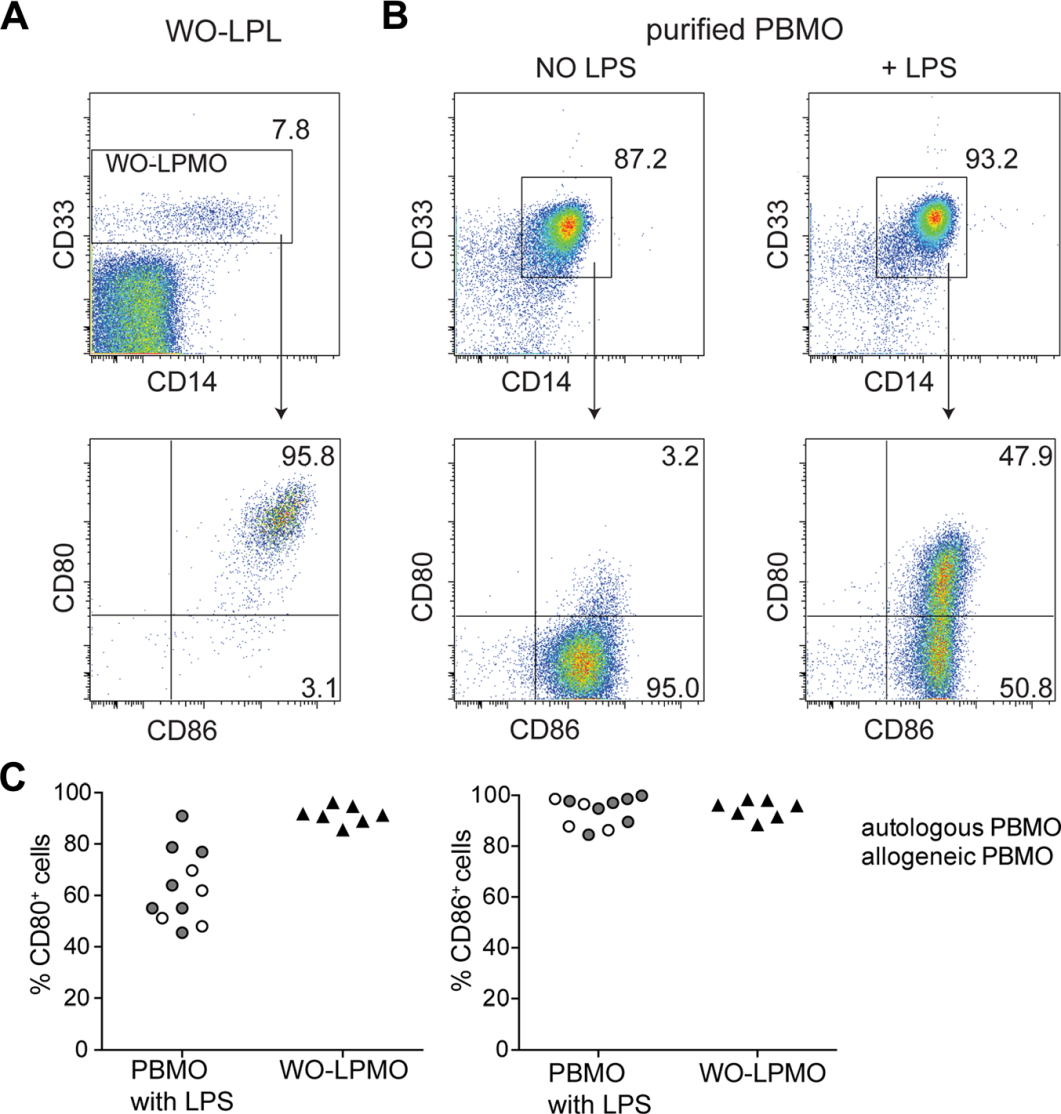
Expression of CD80 and CD86 on WO-LPL and PBMO. (A) Representative FACS plots of WO-LPL harvested after 36 h of organ culture and stained for surface expression of CD33 and CD14 (upper panel). Further, the surface expression of CD80 and CD86 of CD33^+^ WO-LPMO (lower panel) is shown. Numbers in each quadrant indicate %. (B) Peripheral blood monocytes (PBMO) were isolated from autologous PB using magnetic beads and activated with 1 µg/mL LPS for 8 h to induce CD80 expression. Representative FACS plots showing the purity of isolated CD14^+^CD33^+^ PBMO (upper panel) and their expression of CD80 in the absence or presence of LPS activation (lower panel). (C) CD80 (left panel) and CD86 (right panel) surface expression (%) of CD33^+^ WO-LPMO (7 tissue donors) and CD14^+^CD33^+^ PBMO (autologous: PB from 4 of the tissue donors; PB from 4 allogeneic donors).

Because RhuDex® binds to CD80, we ensured the presence of CD80 on immunocompetent cells emigrating from our gut-culture model of general inflammation, following EDTA-mediated loss of the epithelial layer. As shown in Fig. 1(A, C) “Walk-Out” lamina propria myeloid cells (CD66b^−^CD33^+^ WO-LPMO) express high amounts of CD80 and CD86 (% CD80^+^: 91.3 ± 3.5; % CD86^+^: 94.5 ± 3.7). Peripheral blood (PB) leukocytes were used as a control to Walk-Out lamina propria leukocytes (WO-LPL). If possible, PB and WO-LP leukocytes from the same donor were investigated. In some cases, due to logistic reasons, PB leukocytes from different, allogeneic donors were also tested. In contrast to WO-LPMO, peripheral blood monocytes (PBMO) do not express CD80 (Fig. 1B). Therefore, PBMO were activated with 1 µg/mL LPS for 8 h to induce CD80 expression before their introduction into the cultures to test RhuDex® (Fig. 1B, C). To exclude that T cells become activated by LPS, PB leukocytes were split into two fractions for differential treatment of T cells and monocytes before co-incubation. From fraction one, CD14^+^ monocytes were isolated and activated with LPS. Fraction two was placed in culture flasks for 8 h and subsequently the portion of PBL that had not adhered to plastic (non-adherent PBL, including T cells) was harvested. Cell composition and lack of strong T cell pre-activation in non-adherent PBL from allogeneic and autologous donors as well as in WO-LPL are reported in Fig. S1(A, B).

### RhuDex® impacts proliferation of lamina propria and peripheral blood T cells

**Figure 2:**
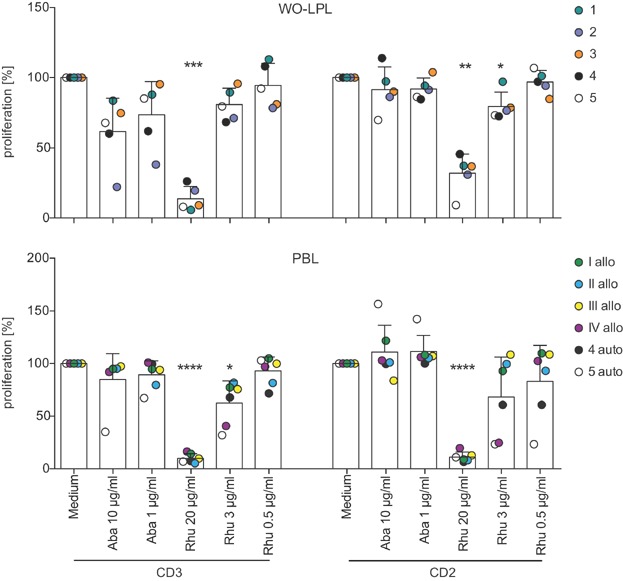
Effect of RhuDex® on proliferation of WO-LP and PB T cells. WO-LPL, or LPS-activated PBMO co-cultured with non-adherent PBL were stimulated using anti-CD3 (OKT3 0.03 µg/mL) and anti-CD2 (M1 0.5 µg/mL, M2 0.5 µg/mL, 3PT 0.3 µg/mL) monoclonal antibodies. RhuDex® and Abatacept were added at the beginning of culture. Proliferation was determined by ^3^[H]-thymidine incorporation at 82–90 h of culture. The mean proliferative responses of each donor in the presence of inhibitors were normalized to the responses without inhibitors (medium, set to 100%). The average 100% response of all donors without inhibitor in WO-LPL corresponds to 33,000 ± 15,700 cpm (anti-CD3) or 22,000 ± 7100 cpm (anti-CD2), and in PBL to 48,000 ± 25,500 cpm (anti-CD3) or 25,000 ± 11,600 cpm (anti-CD2). The upper graph depicts responses in WO-LPL (5 donors, numbered 1–5). The lower graph indicates responses in PBL of 4 allogeneic (allo, numbered I–IV) and 2 donors autologous (auto) to WO-LPL. Data points for each donor are shown in individual colors, and the mean ± SD of all data points in each condition is shown as columns and error bars. *P < 0.05; **P < 0.01; ***P < 0.001; ****P < 0.0001. Aba, Abatacept, Rhu, RhuDex®.

Next, the effect of RhuDex® on the proliferation of lamina propria (LP) T cells was tested. Abatacept, which binds to both CD80 and CD86 was used for comparison. To this end, WO-LPL, which had emigrated from the cultured intestinal mucosa, were stimulated through TCR/CD3, or CD2-receptor using monoclonal antibodies, or left unstimulated (medium control) in the presence or absence of increasing concentrations of RhuDex® and Abatacept. WO-LPL were investigated in parallel with a co-culture of non-adherent PBL and LPS-activated PBMO. Fig. S2 (representative data of one donor) shows that proliferation of T cells in WO-LPL and PBL as detected by ^3^[H]-thymidine incorporation was strongly inhibited by RhuDex® in response to both anti-CD3 or anti-CD2 stimulation. In contrast, Abatacept showed no significant anti-proliferative effect in the tested concentrations. By normalizing the proliferation data from all experiments, we consistently observed, that 20 µg/mL of RhuDex® led to a significant reduction of T cell proliferation in response to anti-CD3 (WO-LPL *P* = 0.0001; PBL *P* = <0.0001) or anti-CD2 stimulation (WO-LPL *P* = 0.0012; PBL *P* = <0.0001) (Fig. 2).

To exclude that the reduction of proliferation in the presence of RhuDex® was due to apoptosis, T cell survival was determined by Annexin V and 7AAD staining after 72 h incubation in the presence of RhuDex® or Abatacept (Fig. S1C). These data confirm that RhuDex® and Abatacept have no negative effect on T cell survival. Furthermore, to ensure that the observed inhibition was not due to unspecific anti-proliferative capacities of RhuDex®, the Jurkat T cell line, lacking CD80 expression, was incubated with RhuDex® (Fig. S1D). RhuDex® and Abatacept did not show any significant inhibitory effect on the proliferation of Jurkat T cells.

### RhuDex® affects cytokine production of lamina propria and peripheral blood T cells

In the inflamed intestine, activated antigen-presenting cells and macrophages expressing CD80 and producing pro-inflammatory cytokines play an important role in modulating T cell differentiation and expansion, leading to an increased secretion of T cell pro-inflammatory cytokines [18,19]. Elevated cytokine levels disturb the balance of gut homeostasis against T cell tolerance, which contributes to continuous mucosal inflammation [20]. We therefore investigated, whether RhuDex® modulates the secretion of proinflammatory cytokines (IL-2, IL-17, IFN-γ, and TNF-α) in the T cell stimulation assay. Representative cytokine concentrations in 24 h culture supernatants of WO-LPL and PBL stimulated by anti-CD3 or anti-CD2 are shown in Fig. S3, analogous to the proliferation data in Fig. S2. Of note is the strong cytokine secretion of WO-LPL, especially in response to anti-CD2 stimulation.

**Figure 3:**
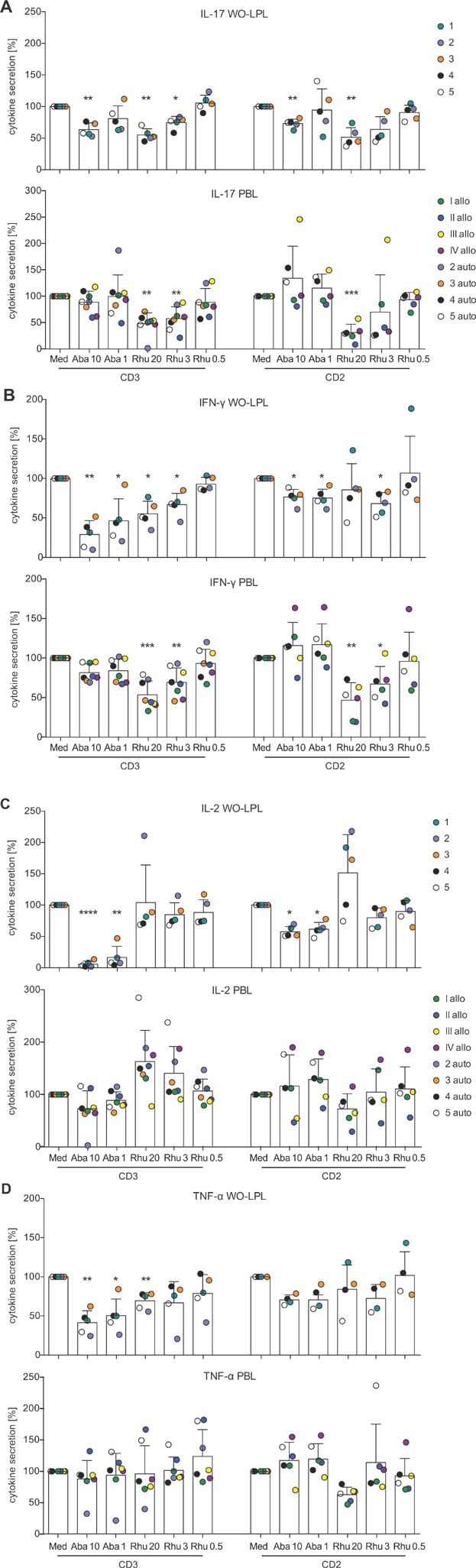
RhuDex® impairs cytokine release of WO-LP and PB T cells. Cytokine concentrations were measured in culture supernatants collected after 24 h of stimulation of WO-LPL, or LPS-activated PBMO co-cultured with non-adherent PBL. RhuDex® and Abatacept were added at the beginning of culture. The mean cytokine responses of each donor in the presence of inhibitors were normalized to the responses without inhibitors (medium, set to 100%). For (A) IL-17, (B) IFN-γ, (C) IL-2, and (D) TNF-α, the upper graph of each panel depicts responses in WO-LPL (5 donors, numbered 1–5). The lower graph indicates responses in PBL of 4 allogeneic (allo, numbered I–IV) and 3–4 donors autologous (auto) to WO-LPL. Data points for each donor are shown in individual colors, and the mean ± SD of all data points in each condition is shown as columns and error bars. *P < 0.05; **P < 0.01; ***P < 0.001; ****P < 0.0001. Med, medium control, Aba, Abatacept, Rhu, RhuDex®, inhibitor concentrations in µg/mL.

Summarizing the responses of five experiments by data normalization shows that RhuDex®, used at concentrations of 3 and 20 µg/mL, significantly inhibited secretion of IL-17 and IFN-γ by WO-LPL and PBL that were stimulated with anti-CD3 (WO-LPL 20 µg/mL: Fig. 3A *P* = 0.0016; Fig. 3B *P* = 0.0107). TNF-α secretion was also decreased in anti-CD3 stimulated WO-LPL, but not PBL, following treatment with RhuDex® (WO-LPL 20 µg/mL: Fig. 3D *P* = 0.0092). With regard to anti-CD2 stimulation, RhuDex® inhibited IL-17 release by WO-LPL and PBL at a concentration of 20 µg/mL, while anti-CD2 stimulated IFN-γ release was only reduced in PBL in a concentration-dependent manner. Secretion of TNF-α induced by anti-CD2 stimulation was not modulated in both cell populations. RhuDex® did not affect IL-2 secretion of anti-CD3 and anti-CD2 stimulated WO-LPL or PBL.

Similar to RhuDex®, Abatacept, when used at a concentration of 10 µg/mL, inhibited IL-17, IFN-γ, and TNF-α secretion by WO-LPL stimulated via anti-CD3. IFN-γ and TNF-α release was also inhibited in the presence of the lower concentration of 1 µg/mL Abatacept. Furthermore, Abatacept inhibited IL-17 and IFN-γ but not TNF-α secretion in WO-LPL in response to anti-CD2 activation. In contrast to RhuDex, IL-2 concentrations in the culture supernatants of anti-CD3 or anti-CD2 stimulated WO-LPL were significantly decreased in the presence of 1 and 10 µg/mL Abatacept. Effects of Abatacept on IL-2 secretion were stronger on anti-CD3 than on anti-CD2 stimulated cells (10 µg/mL: anti-CD3 *P* = <0.0001, anti-CD2 *P* = 0.0343). Importantly and different to RhuDex®, Abatacept did not affect cytokine secretion in PBL under the conditions tested.

### Cytokines are primarily produced by CD4^+^ lamina propria T cells following activation

**Figure 4:**
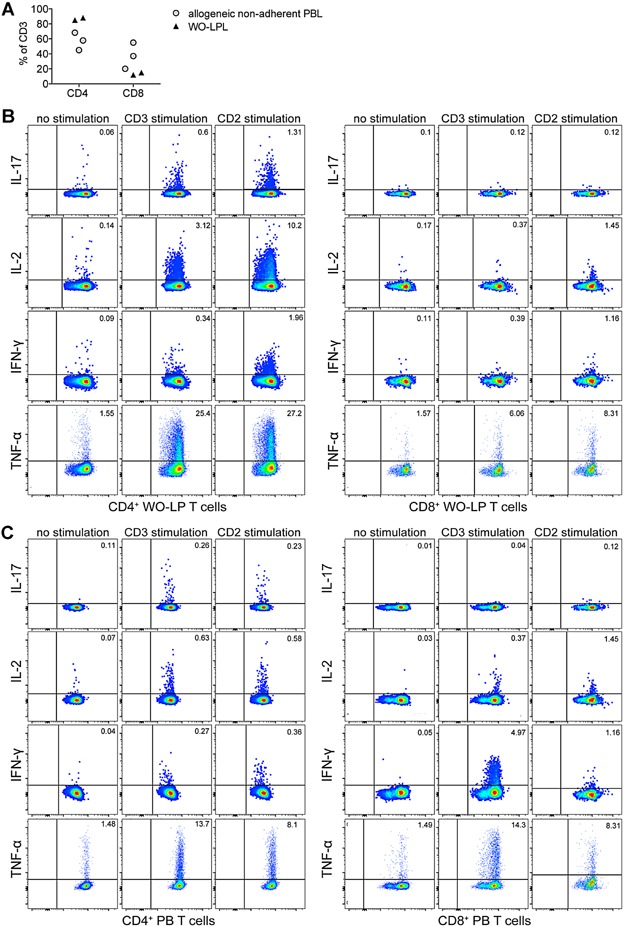
Intracellular cytokine expression of CD4^+^ or CD8^+^ WO-LP and PB T cells after activation. (A) Proportion (%) of CD4^+^ and CD8^+^ T cells among CD3^+^ T cells in PBL (3 allogeneic donors) and WO-LPL (2 tissue donors). (B) Representative dot-plots (donor III) showing the intracellular cytokine expression (IL-17, IL-2, IFN-γ and TNF-α) of CD4^+^ WO-LP T cells (left panel) and CD8^+^ WO-LP T cells (right panel), or (C) of CD4^+^ PB T cells (left panel) and CD8^+^ PB T cells (right panel), as detected in the absence of stimulation, or 6 h of anti-CD3, or anti-CD2 stimulation.

In order to determine which T cell subsets preferentially contribute to the cytokine production and are affected by RhuDex®, intracellular cytokine expression after 6 h of anti-CD3 or CD2 stimulation was determined in WO-LPL and PBL. WO-LP T cells consisted mostly of CD4^+^ T cells (Fig. 4A), therefore, cytokine responses of CD8^+^ WO-LP T cells were at the detection limit, which was especially the case for IL-17. In WO-LPL, CD4^+^ T cells were the primary producers of IL-17, IL-2, and TNF-α, while IFN-γ was expressed by a similar fraction of both CD4^+^ and CD8^+^ WO-LP T cells (Fig. 4B). Also in PBL, IL-17, and IL-2 was expressed more by CD4^+^ T cells, however, IFN-γ was produced by a larger fraction of CD8^+^ T cells (Fig. 4C). Except for TNF-α, both, CD4^+^ and CD8^+^ WO-LP T cells showed stronger cytokine production in response to anti-CD2 stimulation when compared to anti-CD3 activation, which was not observed for PB T cells. Notably, the fraction of IL-2 and IL-17 producing CD4^+^ WO-LP T cells was higher than that of CD4^+^ PB T cells following anti-CD3, and in particular anti-CD2 stimulation. These results correlate with the cytokine secretion seen after 24 h of stimulation (Fig. S3), except for TNF-α, where also monocytes are likely to contribute to the TNF-α detected.

**Figure 5:**
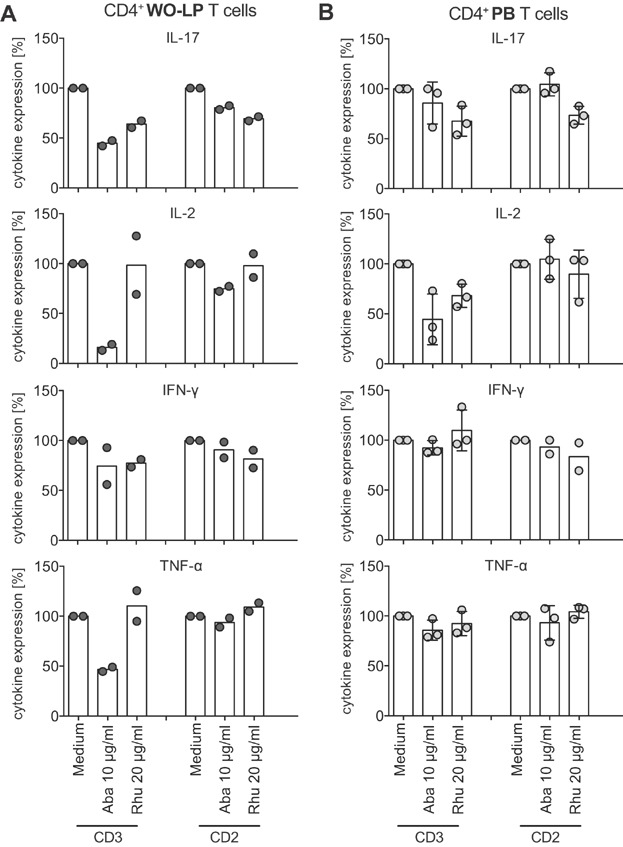
RhuDex® impairs cytokine release of CD4^+^ T cells. WO-LPL and PBL were stimulated with anti-CD3 or anti-CD2 for 6 h and Brefeldin A was added for the last 4 h. The fraction of T cells expressing intracellular cytokines (IL-17, IL-2, IFN-γ, and TNF-α) as gated on CD3^+^CD4^+^ T cells were determined. Shown is the normalized intracellular cytokine expression of (A) CD4^+^ WO-LP T cells (2 tissue donors) and (B) CD4^+^ PB T cells (2 allogeneic donors) in the absence of inhibitors (medium set to 100%) and in the presence of inhibitors (Aba, Abatacept, Rhu, RhuDex®). Data points for each donor are shown in grey circles, and the mean of all data points in each condition is shown as columns.

Because WO-LP T cells were primarily comprised of CD4^+^ T cells, we focused on the modulation of intracellular cytokine expression by RhuDex® compared to Abatacept in CD4^+^ WO-LP and PB T cells (Fig. 5). Again, RhuDex® had the strongest inhibitory effect on IL-17 production in CD4^+^ WO-LP and PB T cells in response to anti-CD3 and CD2 stimulations, similar to the results seen after 24 h in culture supernatants (Fig. 3A). IFN-γ expression, however, was not as strongly affected by RhuDex® in CD4^+^ WO-LP and PB T cells after this shorter 6 h stimulation. Again, Abatacept showed its strongest inhibition on IL-2, IL-17, and TNF-α production in CD4^+^ WO-LP T cells in response to anti-CD3 stimulation (Fig. 5A). Notably, Abatacept also inhibited IL-2 production in CD4^+^ PB T cells following anti-CD3 stimulation for 6 h (Fig. 5B), which contrasts with its lack of effect on IL-2 release by total PB T cells during anti-CD3 stimulation for 24 h (Fig. 3C). This discrepancy may not only be due to time kinetics of IL-2 production, but also due to the lack of effect of Abatacept on CD8^+^ PB T cells (Fig. S4B), which constitute a significant proportion of the total PB T cell population (Fig. 4A).

### Monoclonal CD80 antibody has different effects on the activation of lamina propria and peripheral blood T cells than RhuDex®

Small molecule inhibitors compared to monoclonal antibodies have been shown to target their receptors through different mechanisms [21]. To further compare the effects of the small molecule inhibitor RhuDex® to a monoclonal antibody targeting CD80 only, proliferation of PBL and cytokine release of WO-LPL and PBL in response to anti-CD3 or anti-CD2 stimulations in the absence or presence of a blocking CD80 mAb was examined. First, a CD80 blocking concentration of 5 µg/mL of the mAb was determined to be sufficient (Fig. S5A). The CD80 mAb had no effect on the proliferation of PBL T cells in response to anti-CD3 or CD2 stimulations (Fig. S5B). We further observed, that CD80 blockage by this mAb led to a decrease of IFN-γ secretion in PBL similar to RhuDex®, both in anti-CD3 and CD2 stimulated cells (Fig. S5C). Different to Rhudex®, no inhibitory effect on IL-17 secretion was detected. Specifically in WO-LPL, a reduction of IL-2 release in response to anti-CD3 stimulation, but no other cytokine, was observed in the presence of this CD80 mAb.

## Discussion

Optimal T cell activation and differentiation require co-stimulatory signals. One major co-stimulatory receptor is CD28, which is constitutively expressed on the surface of T cells [22,23]. Ligation of this receptor by its ligands CD80 and CD86 leads to enhanced secretion and stabilization of IL-2 mRNA [24,25], and up-regulation of anti-apoptotic proteins [26] in TCR/CD3 stimulated T cells. While CD86 is constitutively expressed on antigen-presenting cells, CD80 expression is up-regulated following activation of these cells [27]. Functionally, both CD28 ligands play different roles in the effector T cell response [28]. On the one hand, recent data shows that CD80 favorably binds CTLA-4 [29,30] and as a result, provides critical suppression of T cell responses protecting from autoimmune diseases [31,32]. CTLA-4, in contrast to CD28, is up-regulated on activated T cells [33] and serves a regulatory function by inducing T cell anergy and apoptosis [34]. On the other hand in other experimental systems, CD80 blockade led to an inhibition of responses, while anti-CD86 monoclonal antibodies caused exacerbation of disease [35,36]. Importantly, in the setting of IBD, CD80, but not CD86 blockade prevented CD4^+^ T cells with pathogenic potential to induce colitis in mice [8]. Further, a CD80 antagonistic peptide mediated protection against IBD in murine models by reducing Th1 related cytokines [37]. Therefore, the individual contribution of the CD28 ligands in IBD may depend on their functional role in the effector phase of the disease, where CD80 seems to be more important in inducing Th1 responses.

Given this observation, CD80 blockade is an attractive therapeutic strategy for the treatment of intestinal inflammation, for example, in IBD. We therefore tested the effect of RhuDex® (a small molecule that binds human CD80 with low nanomolar affinity, and blocks CD28 and CTLA-4 binding [12]) on the activation of intestinal T cells in a standardized model of general inflammation. We compared its immunomodulatory properties with that of Abatacept, a recombinant fusion protein between the extracellular domain of human CTLA-4 with the Fc part of a human IgG1 [14]. Abatacept has shown good efficacy in treating rheumatoid and juvenile idiopathic arthritis [38,39], however, it has not been found efficacious in human trials in patients with Crohn's disease or ulcerative colitis [40,41].

Considering the fact that Abatacept blocks both CD80 and CD86, whereas RhuDex® does not bind to CD86, it was not surprising to observe different effects of both inhibitors on proliferation and cytokine secretion in response to T cell activation. The cytokines IL-17 and INF-γ in WO-LPL were affected by both inhibitors, with the effect of Abatacept on IFN-γ appearing slightly stronger. In contrast, RhuDex® strongly blocked proliferation of WO-LPL, yet had no effect on IL-2 release, while Abatacept strongly reduced IL-2 secretion, yet had no effect on T cell proliferation. Since Abatacept was not effective in clinical IBD trials, and here we observed a marked IL-2 blockage in the presence of Abatacept in WO-LPL, one could speculate that the presence of IL-2 in the lamina propria of patients with IBD is more important for regulation than inflammation. This view is supported by the fact that IL-2 and IL-2-receptor knockout mice develop spontaneous colitis [42], which is thought to be due to the absence of CD4^+^CD25^+^ T regulatory cells (Treg), dependent on the presence of IL-2 for their suppressive function [43–45]. Treg were detected in the intestinal lamina propria in humans and are thought to be important for normal intestinal immune-homeostasis [46]. In addition to IL-2, also CTLA-4 signals are important for Treg function [47], which may be critical to consider in studies with full CD80/CD86 blockage. Consequently, RhuDex® may be of an advantage in treatment of IBD, because in its presence CTLA-4 can still be engaged by CD86 and sufficient amounts of IL-2 are present in the system, leaving an option for Treg function and maintenance of mucosal immune tolerance. Furthermore, we observed a blockage of peripheral blood T cell proliferation and attenuation of IL-17 and IFN-γ secretion by RhuDex®. This suggests another benefit of RhuDex®, potentially clinically relevant because also T cells from peripheral blood infiltrate intestinal tissue in IBD [48]. Importantly, Rhudex® as a small molecule inhibitor showed a more profound inhibitory effect on PB T cell activation when compared to a CD80 monoclonal antibody, which has previously been shown to block in vitro T cell activation [16]. Similar to Rhudex®, the latter antibody reduced CD3 and CD2-mediated IFN-γ secretion, respectively, in PBL. However, in contrast to Rhudex®, it did not inhibit IL-17 secretion as well as proliferation of PBL in response to these stimuli at the concentrations tested. Furthermore, an effect on T cell specific cytokine production as determined by intracellular FACS staining could not be observed (data not shown). The differential mode of action of both CD80 blocking compounds may be related to different binding characteristics. Additional advantages of RhuDex® are that it can be administered orally and is tolerated well as shown in patients with rheumatoid arthritis [49].

Because WO-LPL consist of a cell mixture, it was determined which T cell subsets are affected by RhuDex® in terms of cytokine production using intracellular staining. We confirmed that CD4^+^ T cells, in the experimental setting of this study, not only produced greater amounts of the cytokines measured, but also RhuDex®, as well as Abatacept, had a greater impact on CD4^+^ T cells in WO-LPL and PBL, than on CD8^+^ T cells. Our observation, that CD4^+^ T cells are more susceptible to CD80 and/or CD86 blockade, is consistent with other studies [32,50,51]. Importantly, it is of relevance to specifically impair CD4^+^ T cell activation in intestinal inflammation, since CD4^+^ T cells predominate in the lamina propria [52], as we also detected in our model. This further indicates, that the T cell specific cytokine results in our 24 h culture supernatants reflect mostly effects on CD4^+^ WO-LP T cells.

An interesting finding of this study was the consistently observed inhibitory effect of CD80 blockade, or CD80/CD86 blockade on T cells when stimulated with anti-CD2 antibodies, particularly in WO-LPL. We hypothesize that CD2, as an alternative pathway to activate T cells [4,5], is an innate mechanism that plays a role in T cell responsiveness in vivo in the intestine. Inhibition of this pathway by CD80- and/or CD86 blockade is not unexpected given that co-stimulation with anti-CD28 has been shown to enhance CD2-induced cytokine secretion in LPL [53]. Our findings demonstrate a role of CD28 as an additive pathway in the response to CD2 stimulation, which may be due to the classic function of CD28 co-stimulation, such as cytokine mRNA stabilization, enhanced T cell proliferation, and induction of anti-apoptotic proteins [24,26]. Apart from blocking CD28 as an additive pathway in the response to CD2 stimulation, RhuDex® may also exert immunomodulatory effects on CD80 expressing cells (dendritic cells, macrophages, or activated monocytes), which in turn could prevent the activation of T cells through regulatory mechanism as has been shown for CTLA-4-Ig, which exerts a direct effect on dendritic cells [54].

In order to investigate the effect of RhuDex® on lamina propria and autologous peripheral blood leukocytes in a standardized setting resembling the in vivo situation, we employed an ex vivo human organ culture model of intestinal inflammation [15]. In this model, T cells have a memory phenotype [13] and lamina propria myeloid cells express CD80, which is in accordance with the high CD80 expression in the intestine of patients with IBD [11]. Notably, CD80 is not expressed on lamina propria myeloid cells isolated by conventional methods using enzymatic digestion of the tissue [55,56], and therefore a different procedure (EDTA treatment) was used, which resulted in CD80 expression on WO-LPMO. Applying our model, we demonstrate that RhuDex® is capable of blocking a human memory T cell response, providing evidence that RhuDex® can be expected to also affect inflammatory responses in vivo. This is consistent with previous studies showing that RhuDex® impairs cytokine secretion and proliferation of rhesus monkey T cells [57]. Further noteworthy, our results show that the intestinal organ culture model represents a useful experimental system applicable in pre-clinical studies evaluating therapeutic compounds for intestinal inflammation.

In conclusion, the strong inhibitory effect of RhuDex® on TCR/CD3- or CD2-mediated lamina propria and peripheral blood T cell proliferation and on IL-17 and IFN-γ secretion, while not affecting IL-2 release, makes it a promising drug candidate for the treatment of chronic intestinal inflammation.

## Supporting Information

**Figure S1**. RhuDex® does not induce apoptosis of PBL, nor inhibits proliferation of Jurkat T cells. **Figure S2**. Effect of RhuDex® on proliferation of WO-LP and PB T cells (representative donor). **Figure S3**. RhuDex® impairs cytokine release of WO-LP and PB T cells (representative donor). **Figure S4**. Cytokine response of CD8^+^ WO-LP and PB T cells in the presence of inhibitors. **Figure S5**. Effects of a blocking CD80 mAb on CD3 or CD2 mediated activation of WO-LPL and PBL. **Table S1**. List of mucosa donors.
