# Supplementary material for: Immunomodulation of human intestinal T cells by the synthetic CD80 antagonist RhuDex®
Source: Immun Inflamm Dis. 2014 Nov 3;2(3):166–80. doi: 10.1002/iid3.34 (PMC4257762; doi:10.1002/iid3.34)
Supplement: Supplementary file 1 — Figure S1. RhuDex® does not induce apoptosis of PBL, nor inhibits proliferation of Jurkat T cells. Figure S2. Effect of RhuDex® on proliferation of WO-LP and PB T cells (representative donor). Figure S3. RhuDex® impairs cytokine release of WO-LP and PB T cells (representative donor). Figure S4. Cytokine response of CD8+ WO-LP and PB T cells in the presence of inhibitors. Figure S5. Effects of a blocking CD80 mAb on CD3 or CD2 mediated activation of WO-LPL and PBL. Table S1. List of mucosa donors. [file iid30002-0166-sd1.pdf]

Immunomodulation of human intestinal T cells by the synthetic CD80 antagonist RhuDex®

SUPPLEMENTARY DATA

Table S1: List of mucosa donors

Subject number	Age	Diagnosis
1	75	Adenocarcinoma of the right hemicolon
2	65	Adenocarcinoma of the rectosigmoid
3	75	Tubulo-villous adenoma of the colon (low grade IEN)
4	63	Adenocarcinoma of the rectosigmoid
5	68	Tubulo-villous adenoma of the colon (high grade IEN)
6	56	Adenocarcinoma of the rectosigmoid
7	66	Adenocarcinoma of the left hemicolon

The mucosa, used in this study, was isolated from colonic tissue and free of pathologic changes as judged by a pathologist.

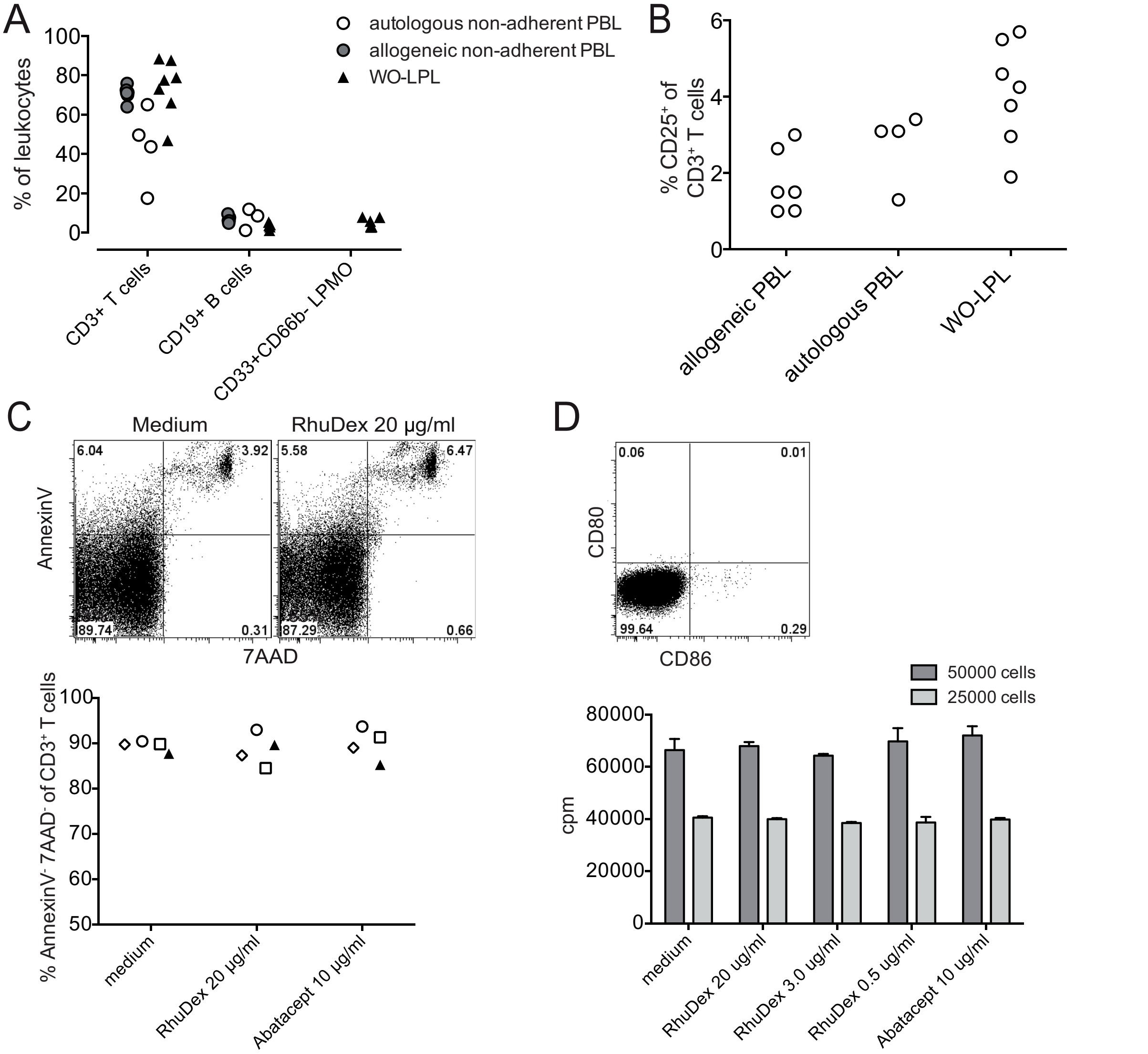

Figure S1: RhuDex® does not induce apoptosis of PBL, nor inhibit proliferation of Jurkat T cells. (A) Proportion (%) of $CD3^+$ T cells and $CD19^+$ B cells in PBL (autologous: PB from 3-4 tissue donors; 5-6 allogeneic donors). Proportion (%) of $CD3^+$ T cells, $CD19^+$ B cells and $CD33^+$ LPMO in WO-LPL (5-7 donors). (B) Proportion (%) of $CD3^+$ T cells expressing CD25 on their surface derived from WO-LPL (7 donors) or PBL (autologous: PB from 4 of the tissue donors; PB from 6 allogeneic donors). (C) Representative FACS plots (upper panel) of an apoptosis assay showing the survival (negative for Annexin V and 7AAD; lower left quadrants) of $CD3^+$ PB T cells. PBL were incubated in the absence (medium only) or presence of 20 µg/ml RhuDex® for 72 hours. Percentages in each quadrant indicate % of $CD3^+$ T cells. The lower panel shows the survival (% of $CD3^+$ T cells) of PB T cells from 3 donors (open symbols) or WO-LP T cells from 1 donor (filled symbol) without or with 20 µg/ml RhuDex® or 10 µg/ml Abatacept. (D) Upper panel: representative dot-plot showing the absence of CD80 and CD86 expression of Jurkat T cells on which the impact of RhuDex® on proliferation was tested. Lower panel: 50,000, or 25,000 Jurkat T cells/well were incubated in the absence (medium) or presence of either 10 µg/ml Abatacept or 0.5, 3, 20 µg/ml RhuDex®. Proliferation was determined by ^{3}[H]-thymidine incorporation after 20 hours of incubation. Mean ± SD of three replicates.

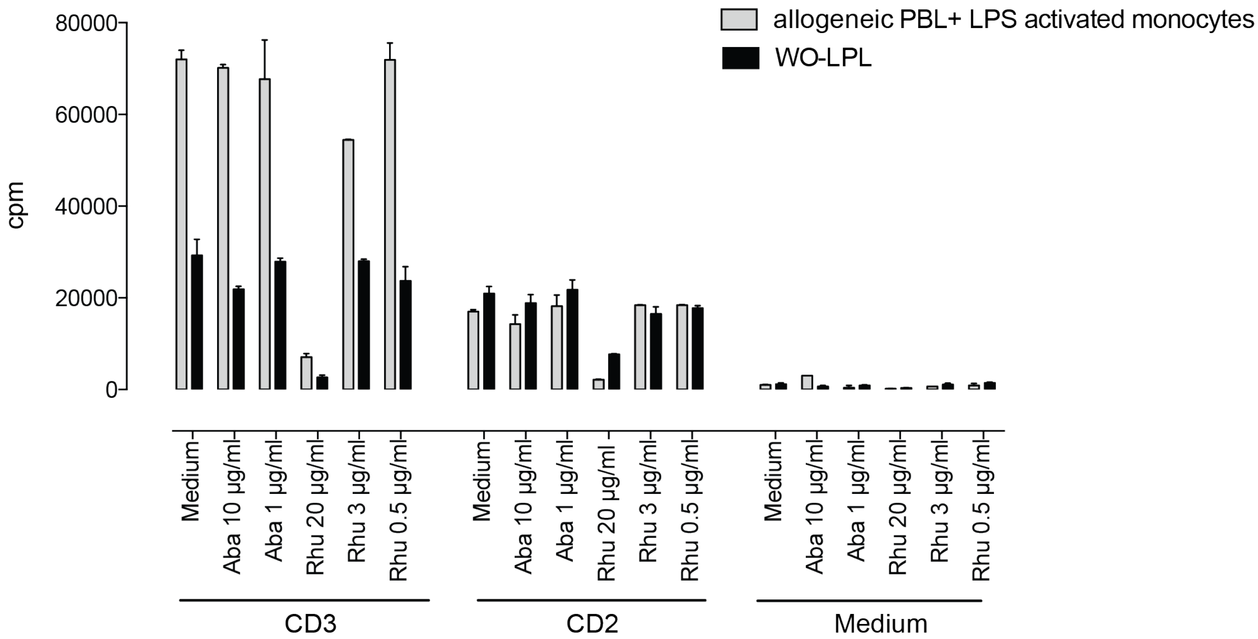

Figure S2: Effect of RhuDex® on proliferation of WO-LP and PB T cells (representative donor). WO-LPL, or LPS-activated PBMO co-cultured with non-adherent PBL were stimulated using anti-CD3 (OKT3 0.03 µg/ml) and anti-CD2 (M1 0.5 µg/ml, M2 0.5 µg/ml, 3PT 0.3 µg/ml) monoclonal antibodies. RhuDex® and Abatacept were added at the beginning of culture. Representative response of T cells from one donor in terms of proliferation as measured by ^{3}[H]-thymidine incorporation at 72-90 hours of culture. Each condition is shown as mean ± SD (counts per minute, cpm) of three technical replicates.

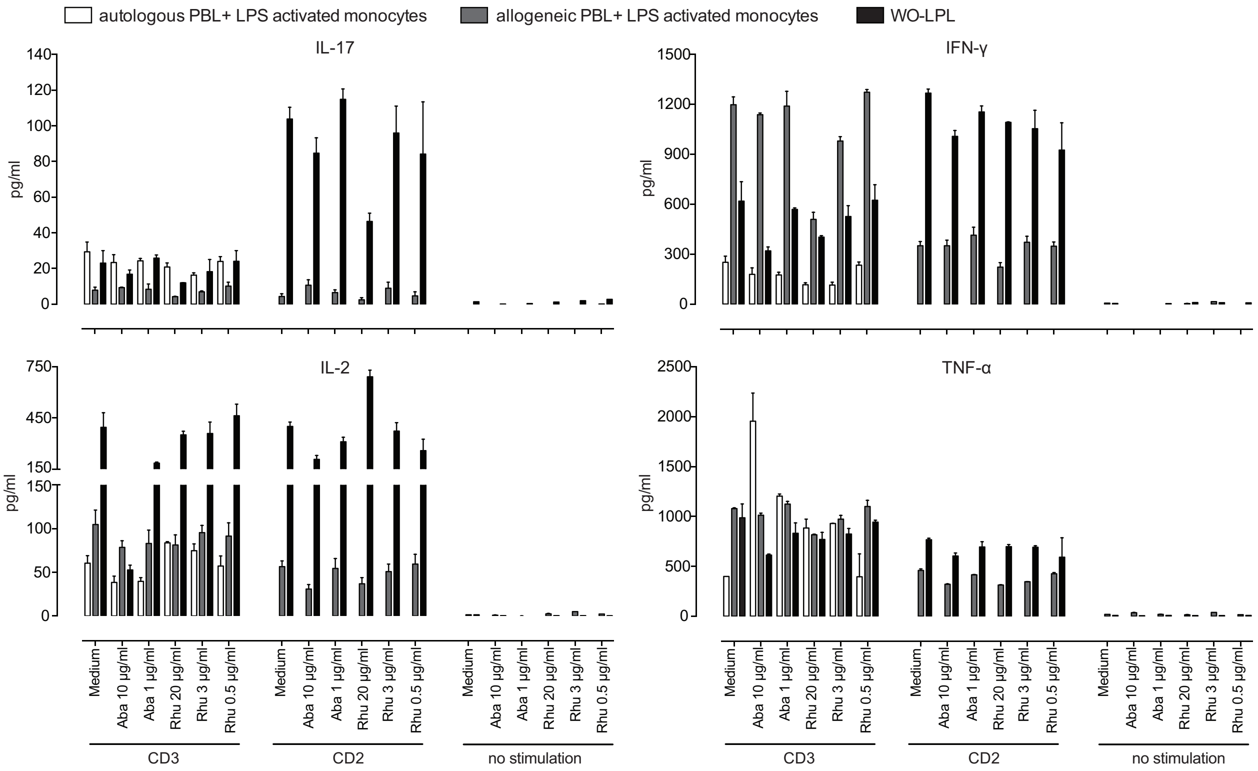

Figure S3: RhuDex® impairs cytokine release of WO-LP and PB T cells (representative donor). WO-LPL, or LPS-activated PBMO co-cultured with non-adherent PBL (autologous: same donor as mucosa, or allogeneic donor) were stimulated using anti-CD3 (OKT3 0.03 µg/ml) and anti-CD2 (M1 0.5 µg/ml, M2 0.5 µg/ml, 3PT 0.3 µg/ml) monoclonal antibodies. RhuDex® and Abatacept were added at the beginning of culture. Corresponding to the proliferative response in Supp Fig. 2, the concentrations (pg/ml) of (A) IL-17, (B) IFN-γ, (C) IL-2 and (D) TNF-α as measured in one representative donor in the supernatants collected after 24 hours of culture are graphed. Each condition is shown as mean ± SD of two technical replicates.

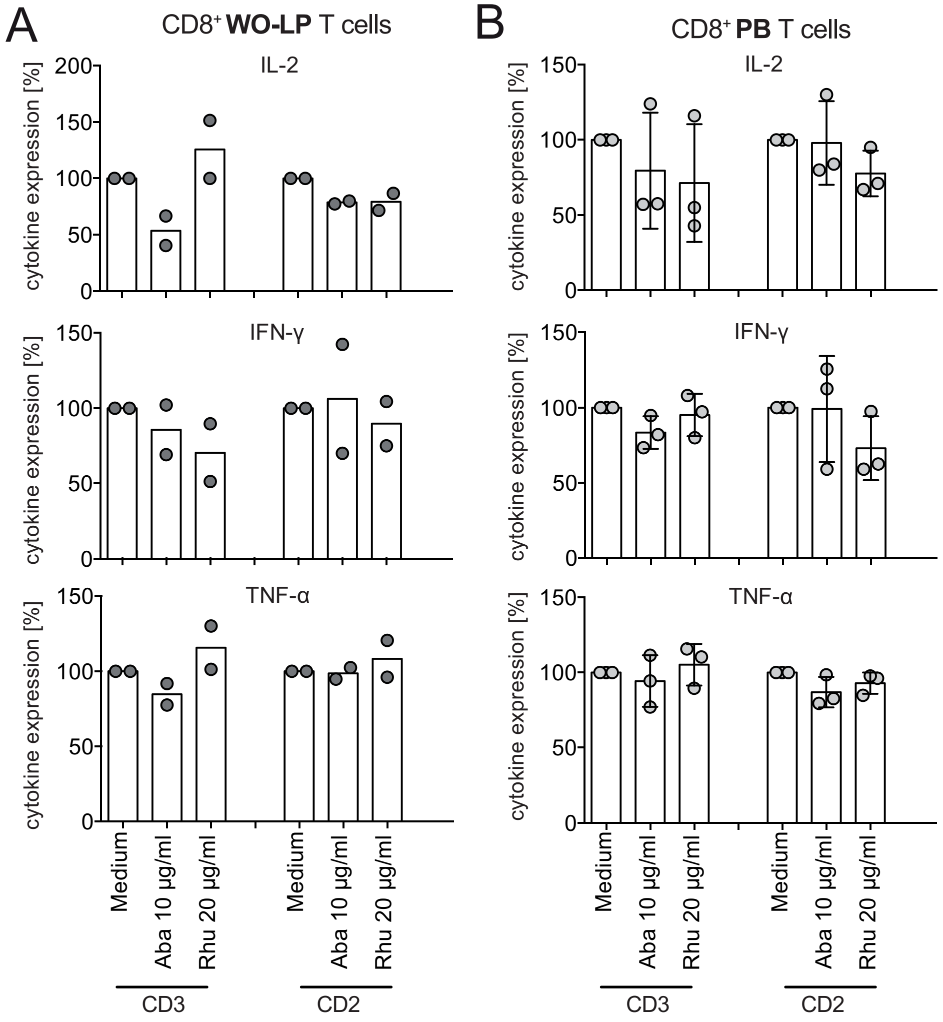

Figure S4: Cytokine response of CD8$^+$ WO-LP and PB T cells in the presence of inhibitors. WO-LPL and PBL were stimulated with anti-CD3 and anti-CD2 for 6 hours and Brefeldin A was added for the last 4 hours. The fraction of T cells expressing intracellular cytokines (IL-2, IFN-γ and TNF-α) gated on CD3$^+$CD8$^+$ T cells was determined. (A) Normalized fraction of intracellular cytokine expressing CD8$^+$ WO-LP T cells (2 tissue donors) and (B) CD8$^+$ PB T cells (3 allogeneic donors) in the absence of inhibitors (medium set to 100 %) compared to the presence of inhibitors (Aba= Abatacept, Rhu= RhuDex®). Data points for each donor are shown in grey circles, and the mean of all data points in each condition is shown as columns.

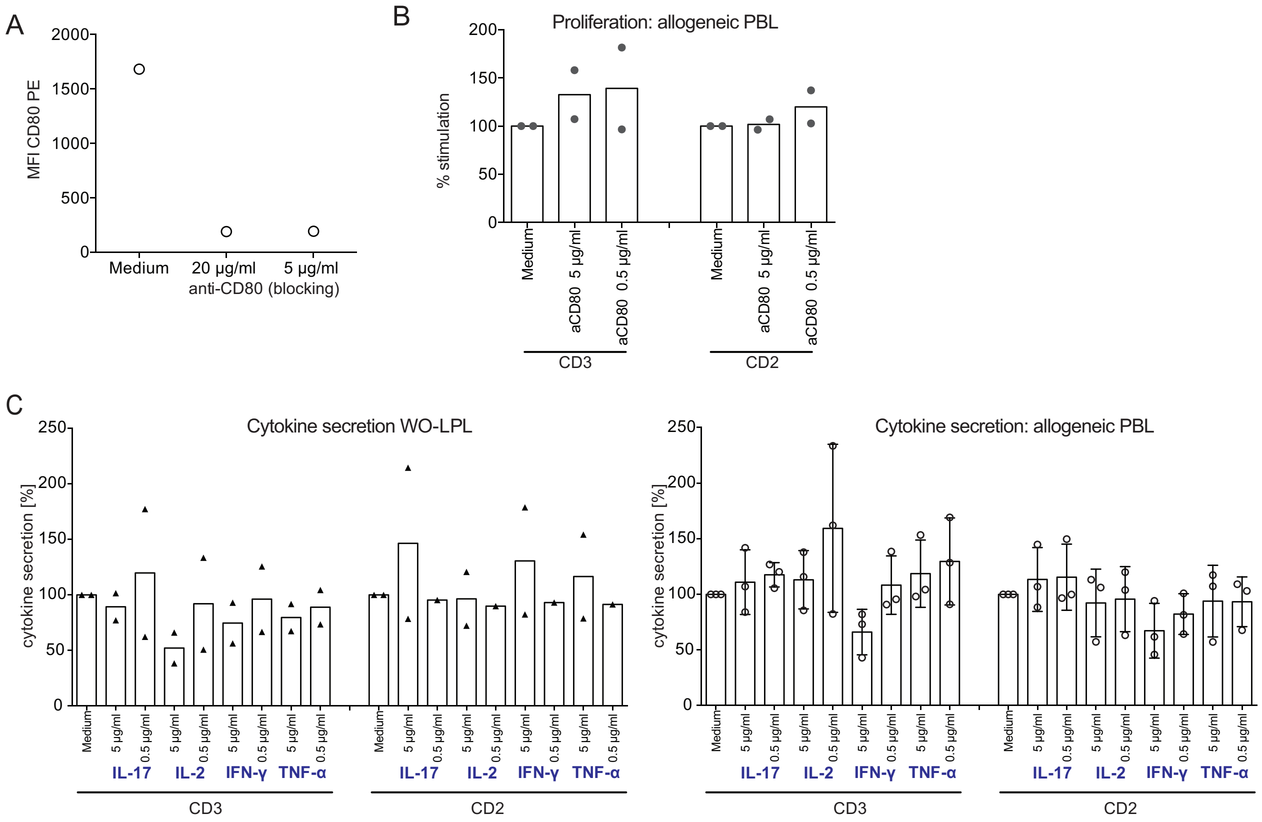

Figure S5: Effects of a blocking CD80 mAb on CD3 or CD2 mediated activation of WO-LPL and PBL. (A) PB monocytes were stimulated with LPS overnight to induce CD80 expression. Subsequently, binding of the blocking CD80 mAb (clone 2D10) was analyzed based on competitive inhibition of a fluorochrome-labeled CD80 antibody on PB monocytes as determined by flow cytometry. Shown is the mean fluorescence intensity (MFI) of CD80 PE staining of $CD14^+$ PB monocytes without (Medium) and after treatment with the blocking CD80 mAb in two concentrations (20 µg/ml and 5 µg/ml). (B) LPS-activated PBMO co-cultured with non-adherent PBL (2 allogeneic donors) were stimulated using anti-CD3 (OKT3 0.03 µg/ml) and anti-CD2 (M1 0.5 µg/ml, M2 0.5 µg/ml, 3PT 0.3 µg/ml) monoclonal antibodies. CD80 mAb was added at the beginning of culture. Proliferation was determined by ^{3}[H]-thymidine incorporation at 90 hours of culture. The mean proliferative responses of each donor in the presence of inhibitors were normalized to the responses without inhibitors (medium, set to 100 %). (C) Normalized cytokine secretion as measured in culture supernatants collected after 24 hours of stimulation in WO-LPL (left panel, 2 donors), or LPS-activated PBMO co-cultured with non-adherent PBL (right panel, 3 allogeneic donors). The mean cytokine responses of each donor in the presence of CD80 mAb (5 and 0.5 µg/ml) were normalized to the responses without inhibitor (medium, set to 100 %). Data points for each donor are shown intriangles or circles, and the mean of all data points in each condition is shown as columns.
